# Neuromarkers of Adaptive Neuroplasticity and Cognitive Resilience Across Aging: A Multimodal Integrative Review

**DOI:** 10.3390/neurolint18010010

**Published:** 2026-01-05

**Authors:** Jordana Mariane Neyra Chauca, Manuel de Jesús Ornelas Sánchez, Nancy García Quintana, Karen Lizeth Martín del Campo Márquez, Brenda Areli Carvajal Juarez, Nancy Rojas Mendoza, Martha Ayline Aguilar Díaz

**Affiliations:** 1 Facultad de Medicina, Universidad Autónoma de Guadalajara, Guadalajara 45129, Mexico; 2 Facultad de Medicina, Universidad Autónoma del Estado de México, Toluca 50180, Mexico; garciaquintananancy@yahoo.com.mx; 3 Facultad de Medicina, Universidad Lamar, Guadalajara 44110, Mexico; karengeminis@live.com.mx; 4 Facultad de Medicina, Universidad Autónoma de Tamaulipas, Victoria 87149, Mexico; brenda_carvajal@live.com.mx; 5 Facultad de Medicina, Benemérita Universidad Autónoma de Puebla, Puebla 72570, Mexico; nancyrjs19@gmail.com; 6 Facultad de Medicina, Universidad Autónoma de San Luis Potosí, San Luis Potosí 78210, Mexico; marthadiaz387@gmail.com

**Keywords:** aging, neuroplasticity, cognitive resilience, neuromarkers, BDNF, fMRI, electrophysiology, neuroimaging, compensation, aging brain

## Abstract

Background: Aging is traditionally characterized by progressive structural and cognitive decline; however, increasing evidence shows that the aging brain retains a remarkable capacity for reorganization. This adaptive neuroplasticity supports cognitive resilience—defined as the ability to maintain efficient cognitive performance despite age-related neural vulnerability. Objective: To synthesize current molecular, cellular, neuroimaging, and electrophysiological neuromarkers that characterize adaptive neuroplasticity and to examine how these mechanisms contribute to cognitive resilience across aging. Methods: This narrative review integrates findings from molecular neuroscience, multimodal neuroimaging (fMRI, DTI, PET), electrophysiology (EEG, MEG, TMS), and behavioral research to outline multiscale biomarkers associated with compensatory and efficient neural reorganization in older adults. Results: Adaptive neuroplasticity emerges from the coordinated interaction of neurotrophic signaling (BDNF, CREB, IGF-1), glial modulation (astrocytic lactate metabolism, regulated microglial activity), synaptic remodeling, and neurovascular support (VEGF, nitric oxide). Multimodal neuromarkers—including preserved frontoparietal connectivity, DMN–FPCN coupling, synaptic density (SV2A-PET), theta–gamma coherence, and LTP-like excitability—consistently correlate with resilience in executive functions, memory, and processing speed. Behavioral enrichment, physical activity, and cognitive training further enhance these biomarkers, creating a bidirectional loop between experience and neural adaptability. Conclusions: Adaptive neuroplasticity represents a fundamental mechanism through which older adults maintain cognitive function despite biological aging. Integrating molecular, imaging, electrophysiological, and behavioral neuromarkers provides a comprehensive framework to identify resilience trajectories and to guide personalized interventions aimed at preserving cognition. Understanding these multilevel adaptive mechanisms reframes aging not as passive decline but as a dynamic continuum of biological compensation and cognitive preservation.

## 1. Introduction

Aging is accompanied by complex neurobiological changes that modify the structure and function of the brain. For decades, this process was primarily described in terms of decline—characterized by synaptic loss, reduced neurogenesis, and progressive cognitive impairment [1,2,3,4]. However, recent evidence challenges this unidirectional view by showing that the aging brain retains a remarkable capacity for adaptation and reorganization, a phenomenon known as adaptive neuroplasticity [1,4,5,6,7].

Neuroplasticity encompasses the brain’s ability to modify neural circuits in response to internal or external stimuli, maintaining homeostasis and optimizing cognitive performance [1,4,8,9,10,11,12]. In older adults, adaptive plasticity can manifest as compensatory recruitment of alternative neural pathways, strengthening of residual synapses, or increased functional connectivity within critical cognitive networks [2,5,6,7,10,13]. These compensatory mechanisms are thought to underlie cognitive resilience, defined as the ability to maintain functional cognition despite age-related structural or molecular changes [13,14,15,16,17,18,19].

Understanding the biological basis of this adaptive capacity has led to increasing interest in neuromarkers—objective indicators of neural processes that reflect the state or efficiency of neuroplastic mechanisms. Neuromarkers can be derived from multiple levels of analysis, including molecular signatures such as BDNF, CREB, and synapsin [20,21,22,23,24,25,26,27,28], neuroimaging correlates linked to network reorganization or connectivity [2,29,30,31,32,33,34,35,36,37,38,39], and electrophysiological measures of cortical excitability and oscillatory dynamics [40,41,42,43,44,45]. Integrating these multimodal biomarkers provides a framework for identifying how some individuals sustain high cognitive performance despite structural brain aging [17,18,19,46,47,48].

This review aims to synthesize current evidence linking neuromarkers of adaptive neuroplasticity to cognitive resilience across aging. By bridging molecular, imaging, and behavioral domains, it seeks to clarify how plasticity-related processes contribute to the preservation of cognition and to highlight emerging biomarkers that may guide early detection and preventive interventions against cognitive decline [18,19,49,50].

## 2. Methods

### 2.1. Literature Search Strategy

This narrative review was conducted following structured and transparent literature selection principles to ensure reproducibility and conceptual rigor. A comprehensive literature search was performed across PubMed/MEDLINE, Scopus, and Web of Science databases. Searches were conducted using combinations of controlled vocabulary terms and free-text keywords related to adaptive neuroplasticity, cognitive resilience, and aging, including but not limited to: “neuroplasticity”, “cognitive resilience”, “brain aging”, “neuromarkers”, “BDNF”, “functional connectivity”, “metabolic plasticity”, “EEG”, “fMRI”, and “PET imaging”. Boolean operators (AND/OR) were applied to refine searches and ensure sensitive coverage.

The search strategy primarily focused on studies published from 2015 onward to capture recent advances in molecular, cellular, and systems-level markers of adaptive plasticity in aging. Earlier seminal studies were selectively included when necessary to provide mechanistic or conceptual context. Inclusion criteria comprised peer-reviewed original research studies and high-quality reviews involving human participants (older adults) and/or validated aging animal models that examined biological, neuroimaging, electrophysiological, or molecular markers associated with adaptive plasticity, cognitive resilience, or preserved cognitive function during aging. Exclusion criteria included studies focused exclusively on advanced neurodegenerative pathology without relevance to adaptive/compensatory mechanisms, and articles lacking sufficient methodological detail or translational relevance. Given the narrative scope of this review, no formal quantitative risk-of-bias assessment was applied; instead, emphasis was placed on consistency, biological plausibility, and convergence across independent studies and on contextual biomarker interpretation (age, cognitive status, and experimental conditions).

### 2.2. Study Selection and Screening

Titles and abstracts were initially screened by two independent reviewers from approximately 480 identified records. Following this screening process, 186 articles were retained for full-text evaluation. Any discrepancies were resolved through consensus. Ultimately, 112 studies were selected based on methodological quality, translational relevance, and contribution to the conceptual framework of adaptive neuroplasticity.

Extracted data encompassed neurotrophic signaling (e.g., BDNF/CREB/IGF-1), glial contributions (astrocytes, microglia, and lactate metabolism), neurovascular and metabolic mechanisms, structural and functional neuroimaging correlates, electrophysiological markers of plasticity (EEG/MEG/TMS measures), behavioral indicators of resilience, and intervention-based evidence including exercise, cognitive training, and neuromodulation.

## 3. Mechanisms of Adaptive Neuroplasticity in the Aging Brain

Neuroplasticity in the aging brain represents a dynamic balance between degenerative processes and compensatory mechanisms that maintain network efficiency [2,5,6,7,8,12,13,51,52]. Rather than a linear decline, aging involves a complex reconfiguration of neural systems driven by molecular, synaptic, and glial adaptations that support cognitive resilience [14,16,17,18,19,20,53] (Table 1).

### 3.1. Molecular Mediators of Adaptive Plasticity

At the molecular level, several signaling cascades regulate synaptic maintenance, dendritic remodeling, and neuronal survival [3,9,12,13]. Among these, BDNF is one of the most consistent indicators of plastic potential [21,22,23,24].

BDNF modulates synaptic strength through TrkB receptor activation, promoting LTP and spine stabilization [21,22,23,24]. Aging is typically associated with decreased BDNF expression; however, individuals with preserved cognitive performance often show maintained or compensatory BDNF upregulation, suggesting its value as a neuromarker of resilience [23,24,54].

The transcription factor CREB acts downstream of neurotrophin signaling, coordinating genes involved in synaptic efficacy and neurogenesis [3,25,28]. Sustained CREB phosphorylation in hippocampus and prefrontal cortex correlates with preserved learning capacity in aging models [3,15,20].

Similarly, IGF-1 and synapsin I contribute to neuronal excitability and vesicle recycling, linking metabolic homeostasis with synaptic adaptability [26,54,55,56,57].

### 3.2. Glial and Metabolic Contributions

Astrocytes and microglia are increasingly recognized as active regulators of neuroplasticity [58,59,60,61,62,63,64,65,66,67].

Astrocytic end-feet modulate neurovascular coupling and supply metabolic support to synapses through the astrocyte–neuron lactate shuttle [58,59,60,61,62]. Lactate acts not only as an energy substrate but also triggers CREB-mediated transcription of plasticity-related genes [28,58,59].

Microglia—traditionally viewed as pro-inflammatory—also mediate synaptic pruning and release trophic factors that facilitate restructuring when maintained in a regulated, non-degenerative state [63,64,65,66,67]. Controlled microglial activation is therefore considered a hallmark of adaptive, rather than pathological, neuroinflammation [63,64,65].

### 3.3. Synaptic and Network-Level Reorganization

At the synaptic level, adaptive plasticity manifests as selective strengthening or weakening of connections that optimize information processing [9,15,16,29,53].

Evidence from aged animal models and human neuroimaging indicates that compensatory remodeling occurs preferentially within hippocampal–prefrontal circuitry, sustaining memory and executive function [17,18,47,68].

Functional MRI studies reveal increased bilateral activation and enhanced cross-hemispheric connectivity, widely interpreted as network-level compensation [19,47,48]. These reorganizations are supported by molecular cascades involving BDNF, glutamatergic receptor trafficking, and cytoskeletal modifications [21,22,23,24,29,55].

### 3.4. Neurovascular and Inflammatory Modulation

Vascular health is fundamental for neuroplasticity, as endothelial dysfunction and reduced perfusion limit energy availability for synaptic activity [32,33,34,63,64].

Endothelial-derived nitric oxide (NO) and VEGF maintain perfusion and angiogenesis in response to neuronal demand [60,61,62,64,65].

Meanwhile, systemic inflammation—when mild and transient—may stimulate adaptive repair via cytokine-mediated neurotrophic signaling, whereas chronic inflammation disrupts these pathways [63,64,65,66,67].

Together, these molecular, glial, and vascular systems form an adaptive plasticity matrix, enabling the aging brain to sustain homeostasis and cognitive efficiency despite structural vulnerability [14,16,17,18,19,20,53].

**Table 1:** Representative molecular and cellular neuromarkers associated with adaptive neuroplasticity in aging [21,22,23,24,25,26,27,28,55,56,57,58,59,60,61,62,63,64,65,69].

Neuromarker	Primary Function	Mechanistic Role in Adaptive Plasticity	Interpretation/Relevance to Cognitive Resilience
BDNF (Brain-Derived Neurotrophic Factor)	Neurotrophic support, synaptic maintenance	Activates TrkB signaling, enhances LTP and dendritic spine growth	Higher BDNF levels correlate with preserved learning and memory in aging; biomarker of plastic potential
CREB (cAMP Response Element-Binding Protein)	Transcription factor regulating plasticity genes	Coordinates gene expression for LTP, neurogenesis, and metabolic adaptation	Sustained CREB phosphorylation reflects long-term adaptive remodeling and preserved cognition
Synapsin I	Synaptic vesicle regulation	Facilitates neurotransmitter release and vesicle recycling	Indicates synaptic efficiency; reduced loss linked to functional compensation
IGF-1 (Insulin-like Growth Factor 1)	Neurotrophic and metabolic regulation	Promotes neuronal survival, myelination, and glucose metabolism	Supports energy–plasticity coupling; low levels associate with frailty and cognitive decline
Astrocytic Lactate	Metabolic coupling between neurons and glia	Acts as both energy substrate and signaling molecule via CREB activation	High astrocytic metabolic responsiveness enhances synaptic maintenance and recovery
GFAP (Glial Fibrillary Acidic Protein)	Structural astrocytic integrity	Reflects astrocyte reactivity and remodeling capacity	Mild elevations may indicate adaptive gliosis; excessive values suggest neurodegeneration
Microglial Activity (regulated state)	Immune modulation and synaptic pruning	Controlled microglial activation releases BDNF and cytokines supporting synapse remodeling	Balanced activation denotes protective neuroinflammation; chronic activation impairs adaptation
VEGF (Vascular Endothelial Growth Factor)	Angiogenesis and vascular remodeling	Stimulates neurovascular coupling and oxygen delivery to active synapses	Elevated VEGF linked to enhanced cerebral perfusion and support of neural plasticity
Nitric Oxide (endothelial)	Vasodilation and perfusion homeostasis	Maintains cerebrovascular reactivity and neurovascular synchronization	Serves as vascular biomarker of metabolic readiness for plastic activity

## 4. Multimodal Neuromarkers of Plasticity

Understanding adaptive neuroplasticity in aging requires integrating information from multiple levels of neural organization [70,71,72]. Beyond molecular mediators, functional and structural imaging, electrophysiological techniques, and emerging biochemical markers provide complementary evidence of how the aging brain reorganizes itself to preserve cognition [17,18,19,47,48]. These multimodal neuromarkers help characterize individual trajectories of resilience and identify compensatory mechanisms invisible to behavioral testing alone [19,73] (Table 2).

### 4.1. Functional and Structural Neuroimaging Markers

Functional MRI (fMRI) studies show that aging produces complex network reorganization rather than uniform decline [17,19,47,48]. Compensatory mechanisms include:

- Bilateral prefrontal activation (HAROLD) [17,47];
- Posterior–anterior shift (PASA) [47,72];
- Dedifferentiation and flexible recruitment [19,47,48].

Resting-state fMRI connectivity within the DMN and FPCN provides sensitive indices of adaptive reconfiguration; preserved or enhanced coupling has been associated with higher cognitive reserve [17,19,73].

Diffusion tensor imaging (DTI) complements these findings by assessing microstructural integrity [74,75,76]. Aging typically reduces fractional anisotropy and increases mean diffusivity, yet resilient individuals show localized preservation of frontoparietal and cingulum tracts essential for long-range compensatory communication [74,75].

Positron emission tomography (PET) extends these insights by revealing molecular correlates of synaptic integrity and metabolic reserve.

The SV2A tracer [^11^C]UCB-J provides a direct measure of synaptic density [30,31], whereas FDG-PET reveals metabolic compensation—where stable cognition coexists with elevated glucose metabolism in prefrontal regions despite hippocampal hypometabolism [32,33,34,35].

### 4.2. Electrophysiological Signatures of Plasticity

EEG and MEG capture temporal dynamics supporting adaptive function. High-frequency gamma oscillations contribute to working memory maintenance [42,43], while theta synchronization supports hippocampal–cortical communication during retrieval [40,44,45].

Cognitively resilient older adults exhibit stronger theta–gamma coupling, reflecting preserved or reconfigured connectivity for cognitive control [44,45].

TMS protocols—including paired associative stimulation and theta-burst stimulation—provide in vivo indices of cortical excitability and LTP-like phenomena [41,77,78,79,80,81]. Aging shows reduced but preserved plasticity responses, indicating partial maintenance of synaptic adaptability [78,79,80,81].

### 4.3. Integrative Multimodal Approaches

Combining modalities yields a more complete characterization of adaptive plasticity [70,71,72]. EEG–fMRI studies demonstrate that increases in network connectivity align with enhanced oscillatory coherence, providing convergent markers of compensation [19,41,47,48,82].

Integrating molecular neuromarkers such as BDNF with neuroimaging or electrophysiology further refines “biomarker constellations” predictive of resilience [17,18,21,22,23,24]. Machine learning tools capable of fusing multimodal signals offer promising avenues for individualized profiling of neuroplastic potential [71,72,73,77].

**Table 2:** Representative multimodal neuromarkers of adaptive neuroplasticity in aging [19,30,31,32,33,34,35,36,37,38,39,40,41,42,43,44,45,46,47,48,74,75,78,79,80,81,82,83,84,85].

Modality	Neuromarker/Measure	Functional Interpretation	Relevance to Cognitive Resilience
fMRI	Bilateral prefrontal activation (PASA pattern)	Recruitment of alternative cortical areas	Reflects compensatory reorganization during task performance
fMRI (resting-state)	DMN–FPCN functional coupling	Network-level integration and efficiency	Predicts preserved executive control and memory
DTI	Fractional anisotropy of frontoparietal tracts	Microstructural integrity of white matter	Indicates maintained long-range connectivity supporting compensation
PET (FDG)	Regional glucose metabolism	Energetic support for network activity	Increased prefrontal metabolism relates to maintained performance
PET ([^11^C]UCB-J)	Synaptic vesicle density (SV2A binding)	Synaptic integrity	Decline in SV2A correlates with reduced plasticity capacity
EEG/MEG	Theta–gamma coupling	Coordination of hippocampal–cortical communication	Enhanced coupling linked to efficient cognitive control
TMS	LTP-like plasticity response (paired associative stimulation)	Cortical excitability and synaptic responsiveness	Serves as in vivo biomarker of preserved plastic potential
Multimodal Integration	Combined EEG–fMRI coherence	Synchronization across scales	Captures individualized adaptive patterns of network resilience

## 5. Cognitive and Behavioral Correlates of Resilience

**Figure 1:**
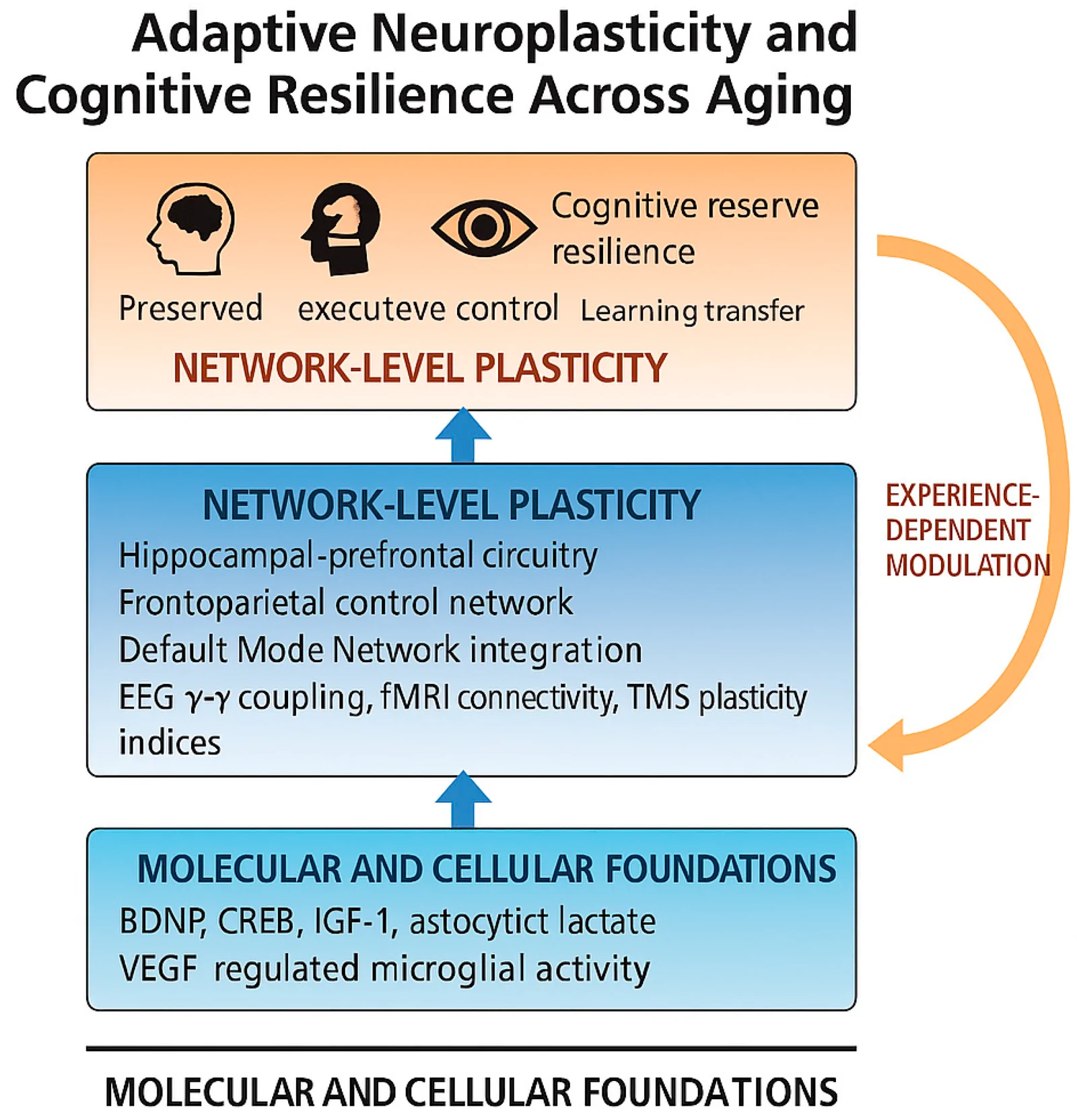
Conceptual model illustrating age-specific mechanisms of adaptive neuroplasticity supporting cognitive resilience, including molecular, glial, neurovascular, and network-level compensatory processes that distinguish resilient aging from youthful plasticity.

Cognitive resilience represents the behavioral expression of adaptive neuroplasticity [17,18,19,47,48,73]. It reflects the ability to maintain or recover cognitive function despite neuropathological burden or structural decline [14,16,17,18,20,53]. This resilience arises from dynamic interactions among neural networks, neuromodulatory systems, and environmental factors that continually reshape brain organization [19,47,48,73]. An overview of the multimodal integration of neuroimaging and electrophysiological neuromarkers is illustrated in Figure 1.

### 5.1. Cognitive Reserve and Compensatory Recruitment

The concept of cognitive reserve (CR) provides a theoretical framework to understand interindividual variability in aging [17,18,19]. CR proposes that education, occupational complexity, and cognitively stimulating activities promote flexible neural strategies [19,44,83,84,86].

Functional neuroimaging demonstrates that older adults with higher reserve engage additional prefrontal and parietal areas during demanding tasks—consistent with HAROLD, PASA, and compensatory recruitment models [17,19,47,48,68,87]. This additional recruitment is a hallmark of adaptive plasticity, enabling preserved performance despite neuronal loss or synaptic inefficiencies [17,19,47,48].

Resilient individuals show identifiable neuromarkers: stronger frontoparietal connectivity, preserved hippocampal activation, and higher resting BDNF levels [17,18,19,21,22,23,24,26,47]. Together, these molecular and systems-level adaptations form an integrated compensatory profile associated with stable cognitive outcomes [17,18,19,47,48].

### 5.2. Neural Efficiency and Reorganization

Beyond compensation, neural efficiency reflects the ability to achieve comparable performance with reduced neural activation [17,19,47,48]. EEG and fMRI evidence shows that resilient older adults exhibit more efficient connectivity patterns, reduced energy expenditure, and strengthened task-relevant synchrony [19,40,41,42,43,44,45,83,84]. This progression—from compensation toward efficiency—suggests consolidation of optimized network architectures [19,44,45,83,84].

Longitudinal data indicate that sustained cognitive engagement, physical activity, and enriched lifestyles support this transition, reinforcing that plasticity remains modifiable throughout the lifespan [19,44,49,50,83,84,86,88,89,90].

### 5.3. Behavioral Markers of Adaptive Plasticity

Behaviorally, adaptive plasticity manifests through preserved executive control, working memory, processing speed, and flexible transfer of strategies to new contexts [49,50,88,89,90,91,92,93,94,95]. Maintenance of attention and inhibitory control correlates with preserved prefrontal synchrony [19,40,41,42,43,44,45,83,84], while efficient encoding and retrieval depend on intact hippocampal–neocortical coupling [17,19,47,48].

Cognitive training, physical exercise, and enriched environments can enhance these adaptive responses, producing measurable increases in BDNF, functional connectivity, and cortical excitability [21,22,23,24,49,50,78,79,88,89,90,91,92,93,94,95,96,97,98].

### 5.4. Toward a Multidimensional Model of Resilience

Integration of neuromarkers with behavioral data shows that resilience is a multilayered process involving cellular maintenance, network-level compensation, and efficient cognitive operations [17,18,19,47,48,73]. Rather than arising from a single biomarker, resilience is better characterized by convergent patterns across biological, functional, and behavioral domains [17,18,19,47,48].

This multidimensional perspective aligns with recent proposals for defining biological signatures of resilience, emphasizing variability and adaptive flexibility as core indicators, rather than markers of dysfunction [18,19,47,48].

Importantly, cognitive resilience in older adults does not reflect a simple preservation of plasticity mechanisms observed in younger individuals, but rather an age-specific adaptive recalibration. At the molecular and cellular levels, resilience is associated with compensatory upregulation of BDNF–CREB signaling despite age-related reductions in baseline expression, enhanced astrocyte-mediated metabolic coupling, and regulated (non-proinflammatory) microglial activation, rather than youthful peak plasticity. At the network level, resilient aging is characterized by a shift from localized efficiency toward distributed and bilateral recruitment, strengthened frontoparietal control network engagement, preserved DMN–FPCN integration, and increased reliance on cross-network coupling to maintain cognitive performance. These alterations contrast with younger adults, in whom plasticity predominantly reflects task-specific specialization, higher synaptic efficiency, and localized network optimization [17,19,47,48,72,73].

## 6. Translational Perspectives and Interventions

Adaptive neuroplasticity provides the biological foundation for prevention and intervention strategies aimed at preserving cognition in aging [17,18,19,47,48,73]. Identifying neuromarkers associated with these adaptive processes has enabled the development of targeted interventions that directly modulate plasticity, bridging experimental neuroscience and clinical applications [29,30,31,32,33,34,35,49,50,66,67,88,89,90,91,92,93,94,95].

### 6.1. Physical Exercise

Aerobic and resistance exercise are among the most robust non-pharmacological enhancers of neuroplasticity [49,50,88,89,90]. Physical activity increases circulating BDNF, IGF-1, and VEGF, which mediate synaptic repair, neurogenesis, and angiogenesis [21,22,23,24,26,28,55,56,57,61,62,63,64]. Exercise also improves neurovascular coupling and cerebral perfusion [31,60,61,62,63], and stimulates hippocampal neurogenesis [21,22,23,24,49,50,55,88,89,90].

Neuroimaging evidence demonstrates enhanced frontoparietal connectivity and improved white-matter integrity after structured exercise programs [42,43,45,46,47,48,74,82,85], changes associated with improved executive function and memory performance [49,50,88,89,90]. Collectively, these findings support exercise as a systemic modulator of adaptive plasticity during aging [49,50,88,89,90].

### 6.2. Cognitive and Multimodal Training

Cognitive training—particularly programs targeting working memory, attention, and executive control—induces measurable neural reorganization [91,92,93,94,95]. Participants often show increased prefrontal activation, strengthened network connectivity, and enhanced theta–gamma coupling [19,37,38,39,40,41,83,84], reflecting improved neural synchrony.

Multimodal interventions that combine cognitive stimulation with physical exercise produce synergistic gains, yielding higher BDNF levels, stronger connectivity, and improved cortical excitability [21,22,23,24,49,50,55,88,89,90,91,92,93,94,95]. These results confirm that behavioral stimulation can function as a biological therapy, inducing measurable changes in neuromarkers [91,92,93,94,95].

### 6.3. Neuromodulation and Pharmacological Support

Non-invasive brain stimulation techniques such as tDCS and rTMS promote cortical excitability and LTP-like plasticity, measurable using TMS-based protocols or EEG indices [38,78,79,96,97,98]. When paired with cognitive engagement, these effects translate into measurable improvements in cognitive performance [78,79,96,97,98].

Pharmacological agents targeting neurotrophin pathways or neurotransmitter systems—including SSRIs and dopaminergic modulators—can potentiate plasticity-related adaptations, although long-term effects in healthy aging require further study [18,23,24,55,99,100].

### 6.4. Clinical Implications and Personalized Interventions

Multimodal neuromarkers support early detection of individuals with low or high plastic potential, enabling precision-based prevention [17,18,29,30,31,32,33,34,35,36,37,38,39,40,41,42,43,54,66,67,70,71,72,74,75,82,99,100,101,102].

For example:

- Low BDNF, reduced functional connectivity, or impaired FA in white matter tracts may signal a need for early intervention [21,22,23,24,42,43,45,46,47,48,55,74,82,85].
- Preserved EEG coherence, metabolic compensation on FDG-PET, or intact network synchrony predict better response to cognitive training or combined interventions [19,29,35,37,38,39,40,41,83,84].

These insights highlight the relevance of biomarker-guided personalization in designing non-pharmacological treatments for cognitive aging [18,54,75,99,100,102].

## 7. Challenges and Future Directions

Despite substantial progress linking neuromarkers to adaptive neuroplasticity, several methodological and conceptual limitations continue to restrict their translational utility [17,18,29,30,31,32,33,34,35,36,37,38,39,40,41,42,43,54,66,67,70,71,72,74,75,80,81,82,99,100,101,102,103,104]. Fully leveraging multimodal biomarkers for predicting and enhancing cognitive resilience will require overcoming these barriers [19,44,45,46,47,48,54,83,84,85,86,102,105,106,107].

### 7.1. Methodological Limitations

A major limitation is that most studies examining neuromarkers of plasticity remain cross-sectional, preventing causal inferences about how molecular or functional changes unfold over time [29,30,31,32,33,34,35,36,37,38,39,40,41,42,43,66,67,74,75,82,91,92,93,94,95]. Longitudinal and multimodal designs—integrating molecular assays, neuroimaging, EEG/MEG, and TMS-based assessments of cortical plasticity—are needed to capture the temporal dynamics of aging-related reorganization [17,18,29,30,31,32,33,34,35,36,37,38,39,40,41,42,43,54,66,67,72,74,75,77,78,79,80,81,82,96,97,98,99,100,101,102,103,104,108,109].

Sample sizes are often modest and heterogeneous, hindering generalizability across populations [17,18,54,70,71,72,99,100,101,102]. Additionally, the lack of standardized acquisition protocols—such as discrepancies in plasma vs. serum BDNF, variability in resting-state vs. task-based fMRI, or non-uniform EEG preprocessing—introduces significant inconsistency [21,22,23,24,29,30,31,32,33,34,35,37,38,39,40,41,42,43,55,66,67,74,75,82].

### 7.2. Integration Across Biological Scales

Many studies examine molecular, network, or behavioral markers in isolation rather than as components of an integrated biological system [17,18,29,30,31,32,33,34,35,36,37,38,39,40,41,42,43,54,66,67,70,71,72,74,75,82,99,100,101,102]. Understanding adaptive neuroplasticity requires multiscale frameworks linking molecular cascades to network remodeling and cognitive outcomes [17,18,45,46,47,48,54,72,75,85,99,100,101,102].

Advanced analytical tools—including connectomics, computational modeling, and machine learning—are promising for identifying latent multimodal patterns predictive of resilience [54,74,75,82,102,105,106,107].

### 7.3. Individual Variability and Personalization

Aging trajectories show wide variability driven by genetics, lifestyle, vascular health, and environmental exposures [17,18,19,44,54,70,71,72,83,84,86,99,100,101,102]. Future neuromarker research must shift toward personalized profiling, integrating composite indices such as BDNF + connectivity + EEG coherence + cortical excitability [19,21,22,23,24,37,38,39,40,41,45,46,47,48,55,80,81,83,84,85,103,104].

Such individualized “plasticity fingerprints” could identify which adults are most likely to benefit from exercise, cognitive training, neuromodulation (TMS/tDCS), or combined interventions [49,50,78,79,80,81,88,89,90,91,92,93,94,95,96,97,98,103,104].

### 7.4. Ethical and Translational Considerations

As neuromarkers move closer to clinical application, ethical challenges emerge concerning predictive labeling, privacy of neurobiological data, and equitable access to high-cost technologies [17,18,54,70,71,72,99,100,101,102]. Translational limitations also persist: many biomarkers require specialized equipment, are expensive, or are not feasible in community settings [29,30,31,32,33,34,35,36,37,38,39,40,41,42,43,66,67,74,75,82].

A key future objective is developing simplified, low-cost, non-invasive neuromarker tools—such as portable EEG, blood-based biomarker assays, or rapid cortical plasticity screens using low-intensity TMS [19,37,38,39,40,41,77,78,79,80,81,83,84,96,97,98,103,104,108,109].

### 7.5. Future Perspectives

Future research should prioritize:

- Multicenter longitudinal studies integrating molecular, imaging, electrophysiological, and behavioral datasets [29,30,31,32,33,34,35,36,37,38,39,40,41,42,43,66,67,74,75,80,81,82,91,92,93,94,95,103,104].
- Validation of composite biomarker panels across diverse populations and risk profiles [17,18,54,68,70,71,72,73,76,87,99,100,101,102,105,106,107,110,111].
- AI-driven multimodal fusion, enabling predictive signatures of adaptive plasticity [54,68,73,74,75,76,82,87,102,105,106,107,110,111].

The convergence of neuroscience, bioinformatics, neuromodulation, and geriatric medicine promises a new era of precision cognitive aging, where interventions are tailored using individualized biomarker-informed profiles rather than chronological age alone [17,18,45,46,47,48,54,68,70,71,72,73,76,80,81,85,87,99,100,101,102,103,104,105,106,107,110,111].

## 8. Discussion

The growing evidence summarized in this review supports a paradigm shift in the understanding of brain aging—from a model of progressive decline to one of dynamic adaptation [2,3,4,5,6,7,8,11,12,13,51,52]. Rather than a passive loss of neural resources, the aging brain displays a remarkable ability to reorganize its structure and function to preserve cognition [15,16,20,45,46,47,48,53,70,71,72,85]. This adaptive neuroplasticity, underpinned by molecular, glial, and vascular mechanisms, constitutes the biological substrate of cognitive resilience [21,22,23,24,25,26,27,28,31,55,56,57,58,59,60,61,62,63,64,65,69].

Importantly, neuromarker interpretation in aging should not rely on a simplified linear framework in which higher values are automatically equated with adaptive neuroplasticity or preserved neural health [25,26,56,57]. In older adults, increases in neuromarkers such as BDNF expression, functional connectivity strength, or regional metabolic activity may reflect compensatory responses to declining efficiency in downstream signaling pathways rather than enhanced function per se [27,28,58,59]. Evidence from neuroimaging and molecular studies indicates that such compensatory upregulation may coexist with reduced network efficiency, altered excitation–inhibition balance, or increased energetic cost during cognitive performance [60,61,62,63].

In addition, aging-related neuromarkers should not be interpreted independently of age range, cognitive status, or measurement context. Neuroimaging and electrophysiological metrics obtained during resting-state conditions may index baseline network organization, whereas task-based measures more directly reflect compensatory recruitment or neural efficiency under cognitive demand [32,33,34,35,36]. Importantly, these patterns vary across the aging spectrum, with younger-old adults often exhibiting flexible compensatory engagement, while older-old individuals may show overactivation associated with reduced performance or increased neural cost [37,38,39,40,41].

Thus, the functional meaning of a given neuromarker is context-dependent and influenced by age range, cognitive status, task demands, and interactions with other biological signals, including inflammatory and vascular factors [64,65,66,69]. As a result, similar neuromarker profiles may carry fundamentally different implications in cognitively resilient older adults compared with individuals at risk for cognitive decline, underscoring the need for cautious and integrative interpretation when evaluating biomarkers of adaptive plasticity in aging populations [29,30,31,67].

However, the heterogeneity of findings across studies underscores that neuroplasticity is not uniformly beneficial. Some compensatory activations may reflect inefficiency rather than resilience, particularly when overactivation of frontal or parietal networks accompanies declining cognitive performance [17,45,46,47,48,72,85,101]. Disentangling adaptive from maladaptive reorganization therefore remains a major conceptual challenge. Future research should integrate longitudinal designs and mechanistic approaches to distinguish compensatory recruitment that sustains function from neural responses that precede cognitive exhaustion [17,18,29,30,31,32,33,34,35,36,37,38,39,40,41,42,43,54,66,67,70,71,72,74,75,82,99,100,101,102].

Another critical point concerns the bidirectional relationship between behavior and biology. Lifestyle factors—such as physical activity, intellectual engagement, and social interaction—can modulate neurotrophic signaling, network organization, and metabolic efficiency [21,22,23,24,49,50,55,88,89,90,91,92,93,94,95], thereby creating a feedback loop between experience and brain biology. Conversely, molecular deficits, including reduced BDNF availability or impaired neurovascular coupling, may constrain the effectiveness of behavioral interventions [21,22,23,24,25,26,27,28,55,56,57,60,61,62]. This interplay highlights the necessity of multilevel models that integrate molecular, network, and behavioral dimensions to fully capture the determinants of cognitive resilience [17,18,45,46,47,48,54,72,75,85,99,100,101,102].

Beyond biological factors, environmental and experiential determinants play a critical role in shaping adaptive neuroplasticity and cognitive resilience in aging. Educational attainment, occupational complexity, physical activity, and sustained cognitive engagement have been consistently associated with more efficient network organization, preserved functional connectivity, and modulation of neurotrophic signaling pathways in older adults [21,22,23,24,49,50,55,88,89,90,91,92,93,94,95]. These factors contribute to cognitive reserve, influencing how neural systems respond to age-related stressors and modifying the functional expression of neuromarkers observed in neuroimaging and molecular studies.

Consequently, similar neuromarker profiles may therefore reflect distinct underlying mechanisms depending on an individual’s environmental background and life-course exposures. For example, increased functional connectivity or metabolic activity may support resilience in individuals with higher cognitive reserve, while representing compensatory strain or inefficiency in less enriched contexts [17,18,29,30,31,32,33,34,35,36,37,38,39,40,41,42,43,54,66,67,72,74,75,82,99,100,101,102]. Integrating environmental determinants into neuromarker-based models is thus essential for accurate interpretation and for advancing precision approaches to cognitive aging.

Importantly, resilience should not be conflated with resistance to aging, but rather conceptualized as adaptive recalibration in response to age-related biological change [17,18,54,70,71,72,99,100,101,102]. The presence of preserved or reorganized neural networks does not imply the absence of pathology, but instead reflects the engagement of compensatory mechanisms that maintain functional homeostasis [17,45,46,47,48,72,85,101]. Integrating multimodal neuromarkers within this framework enables a more nuanced view of brain aging—one characterized not by inevitable loss, but by the dynamic balance between degeneration and adaptation. This perspective aligns with the emerging paradigm of precision cognitive aging, which emphasizes individualized trajectories shaped by biological, environmental, and experiential factors [19,29,30,31,32,33,34,35,36,37,38,39,40,41,42,43,44,45,46,47,48,54,66,67,74,75,82,83,84,85,86,102].

## 9. Conclusions

Adaptive neuroplasticity represents a central mechanism through which the aging brain preserves cognitive function and reorganizes itself in response to biological and environmental demands [5,6,7,11,12,13,17,18,51,52,54,70,71,72,99,100,101,102]. Rather than a passive trajectory of decline, aging reflects a continuous interplay between degeneration and compensation—an evolving equilibrium shaped by molecular, glial, vascular, and network-level processes [15,16,17,18,19,20,27,28,31,44,45,46,47,48,53,54,58,59,60,61,62,63,64,65,69,70,71,72,83,84,85,86,99,100,101,102].

Neuromarkers such as BDNF, CREB, IGF-1, VEGF, indices of synaptic integrity, functional connectivity, white-matter structure, and cortical excitability provide quantifiable windows into these adaptive mechanisms [19,21,22,23,24,25,26,27,28,37,38,39,40,41,55,56,57,58,59,60,61,62,83,84]. Importantly, physiological measures of plasticity obtained through non-invasive brain stimulation—including TMS, tDCS, paired associative stimulation, and theta-burst paradigms—offer robust biomarkers of cortical adaptability across the lifespan [77,78,79,80,81,96,97,98,103,104,108,109]. These neurophysiological signatures complement molecular and imaging-based neuromarkers, strengthening multimodal models of aging [78,79,96,97,98,103,104].

Integrating neuromarkers across biological scales—from cellular metabolism and neurotrophic signaling to network reorganization and behavioral performance—provides a unified framework to understand how cognitive resilience emerges from plasticity [17,18,29,30,31,32,33,34,35,36,37,38,39,40,41,42,43,45,46,47,48,54,66,67,70,71,72,74,75,82,85,99,100,101,102]. Evidence that cortical plasticity varies across individuals, influenced by genetic, lifestyle, and neurobiological factors, further reinforces the need for personalized models of aging [80,81,103,104].

The convergence of molecular neuroscience, multimodal imaging, electrophysiology, and cognitive science demonstrates that plasticity and vulnerability coexist across the lifespan [17,18,45,46,47,48,54,70,71,72,85,99,100,101,102]. The future of aging research will depend on the development of composite biomarker signatures, incorporation of TMS- and EEG-based plasticity metrics, and implementation of personalized interventions guided by individual plasticity fingerprints [19,44,45,46,47,48,49,50,54,74,75,78,79,80,81,82,83,84,85,86,88,89,90,91,92,93,94,95,96,97,98,102,103,104].

Recognizing neuroplasticity as a lifelong capacity reframes aging not as a trajectory of inevitable decline, but as a dynamic continuum of biological adaptability and cognitive resilience [17,18,19,44,45,46,47,48,54,68,70,71,72,73,76,80,81,83,84,85,86,87,99,100,101,102,103,104,105,106,107,110,111].

## Data Availability

No new data were created or analyzed in this study. Data sharing is not applicable.
